# Tourist loyalty and mosque tourism: The case of the Mosque-Cathedral in Córdoba (Spain)

**DOI:** 10.1371/journal.pone.0242866

**Published:** 2020-12-01

**Authors:** Virginia Navajas-Romero, Ricardo David Hernández-Rojas, Amalia Hidalgo-Fernández, Juan Antonio Jimber del Rio

**Affiliations:** 1 Department of Statistics, Econometrics, Operational Research, Business Organization and Applied Economics, University of Cordoba, Córdoba, Spain; 2 Department Agricultural Economics, Finance, and Accounting, University of Cordoba, Córdoba, Spain; Univerza v Mariboru, SLOVENIA

## Abstract

Loyalty is important in the tourism sector since tourists are the key to returning to a destination or recommending it, which is a determining factor in the management of tourist sites. The tourism of Mosques, is a contextualized tourism within religious and cultural tourism. This research aims to analyze the loyalty of tourists of Islamic origin in the Cathedral Mosque of Cordoba. Unlike previous studies, this research adopts a comprehensive approach by considering cultural factors in the analysis of loyalty of Islamic tourists in mosque tourism. The methodology used in this study was a structural equation model with a partial least squares (PLS) analysis. The sample is made up of 262 tourists of Islamic origin at Cordoba Cathedral Mosque. This model does not correspond to factors identified by the previous literature, which adopts an religious perspective of Islamic tourists in mosque tourism. The methodology used in this study was a structural equation model with a partial least squares (PLS) analysis. The sample is made up of 262 tourists of Islamic origin in Cordoba Cathedral Mosque. This model does not correspond to factors identified by the previous literature, which adopts an religious perspective.

## Introduction

In recent years the relationship of tourism with Islam has been the central point of academic research [1, 2]. Mosques are heritage sites which unify key factors in society, such as religion and culture, representing one of the most important and least studied multidisciplinary research fields of the decade [3]. Tourism at mosques is a very specialized type of cultural and religious tourism for Islamic heritage that is included in heritage tourism [4] and it relates to Arabic heritage in heritage tourism. Cultural tourism encompasses a relatively large number of tourists and is growing at an unprecedented pace (OMT, 2015). This type of tourism has gained importance in recent years [5], and tourism at mosques has been investigated previously [6]. In Europe, Spain is a destination for tourists interested in Islamic legacy [7, 8]. The increase in the number of Islamic tourists wishing to connect with their historical legacy is a growing opportunity for tourism in Europe and, consequently, appropriate marketing strategies for this type of tourism have been proposed [5]. The Muslim travel market remains one of the fastest growing sectors in the world with an estimated 140 million Muslim visitors worldwide in 2018, compared to 131 million in 2017 [9]. Tourism at mosques has been recognized as a valid part of this growing market [10] and correct marketing is of great importance [7]. Muslim tourists have some characteristic demands [11] which make Islamic-Muslim tourism at destinations a challenge for tour operators [12].

Sharia Law defines the rules, guidelines and rituals that must be followed when one is a Muslim. Some people misunderstand Muslim communities and think that Islam means terrorist attacks or terrorist groups. These groups, while not being representative of Islam on the whole, are groups which fight for an Islamic State with a violent and extreme interpretation of Sharia law. The consequence of these actions has been the association of Islam with anti-Western violence and ideologies. Because of this, Muslims in certain areas are misjudged and even feared. This has a wide-ranging global impact on tourism and tourists of Muslim origin. In fact, Western tourists prefer to select non-Muslim destinations, while Muslim tourists prefer to travel regionally or resort to neighboring countries [13].

The Mosque-Cathedral in Cordoba is a unique heritage site for several reasons. Firstly, it represents the union of three cultures, Muslim, Jewish and Christian which cannot be seen at many other sites [14]. Secondly, the Mosque-Cathedral is recognized as the second most important temple in the Muslim world, with only the Mecca being more important [15]. Thirdly, the magnificent conservation work of the building [16] and finally as it is the second most visited heritage site in Spain and is included in the “World Heritage Cities” tours [17]. Building at the Mosque-Cathedral in Cordoba began in the year 786. It had continuous improvements during the different periods of the Caliphate of Cordoba. In 1238, after the Reconquest by the Castilian kings, it was consecrated as a cathedral, and in 1523 a Renaissance Plateresque style basilica was built inside the mosque. The mosque had some extensions added in the 16th century [18]. In addition, the Mosque-Cathedral makes Cordoba the first city to have four UNESCO World Heritage sites. In fact, the Mosque-Cathedral is one of the most visited cultural heritage sites, both in Cordoba and Spain. In 2019 it had 2,079,160 tourist visits, and an average growth of visits of 6.5% during the last 10 years (Observatorio de turismo de Córdoba, 2019). The loyalty of the tourist of Islamic origin is special to other types of tourists, the reason for agreement Wardi, Abror and Trinanda (2018) [19] is that for this type of tourism it is essential the implementation of halal and specialized marketing strategies as a key factor to loyalty to this client, since this client prioritizes these factors. Islamic tourists are well-ordered to follow Islamic schooling which directly and indirectly impact on their decision concerning tourism plan.

Satisfying customer needs is a high priority task for tour service providers and they have been seen to be making great efforts to meet the needs of their clientele [20]. Tourists are offered activities which encourage them to enjoy the history and culture of the destination [21]. Visiting a historic mosque may be for religious, cultural or even both reasons [22]. Consequently, studies on tourism at destinations with mosques include about the religious aspects [23, 24] and also about the cultural aspects [25, 26]. There are numerous studies that conclude that mosques are sacred sites which can offer a unique tourist experience [27]. It is therefore important for managers of heritage tourism to take advantage of tourist loyalty, satisfaction and expected value [28]. This study investigates these concepts in terms of the number of visits to the Mosque-Cathedral to help improve the management of tourism [29].

The American Customer Satisfaction Index is a proven and tested model which has been used in studies in the literature (ACSI) [30–33]. The model has a solid basis that can be successfully adapted to different areas in order to analyze the satisfaction and loyalty of the users of a product or service. Customer satisfaction is measured on a scale of 1 to 10, with 1 meaning the customer is very dissatisfied and 10 very satisfied. Customer expectation is also measured on a 1 to 10 scale, with 1 meaning the product or service does not meet expectations and 10 meaning that it exceeds expectations. Quality control associations, research groups and universities from countries such as India, Saudi Arabia, Singapore, Dubai, Kuwait, South Africa, Honduras, Puerto Rico and Colombia implement quality control and satisfaction systems. The literature includes similar user satisfaction measurement systems such as "Swedish Customer Satisfaction Barometer" (SCSB) [34], “Norwegian Customer Satisfaction Barometer” (NCSB) [35], “European Customer Satisfaction Index” (ECSI) [36], “Hong Kong Customer Satisfaction Index” (HKCSI) [37]. The model has been used to measure customer loyalty in different areas, such as psychology [38], satisfaction with university studies [39] or rural development [40]. It has also been widely used to study the tourism sector [41, 42].

The aim of this article is to contribute to existing literature in two ways. Firstly, it presents an assessment of tourism at mosques, and then provides ideas for the administration and management of heritage sites and cultural tourism of Islamic origin. The information available about the complexity and diversity of tourism at Islamic heritage sites is refined with a theoretical model in a case study that analyzes the loyalty of tourists from groups with Islamic heritage. Misguided management and marketing decisions could promote negative aspects such as over-exploitation, creating hierarchies and misunderstandings [43]. On the other hand, this type of tourism also has strengths and opportunities for economic diversification and historical research, as well as the acceptance and dissemination of interculturality. The visitors’ satisfaction and assessment of the perceived value of the Mosque-Cathedral and the destination are used to make useful recommendations to the different public and private bodies that are interested in promoting, disseminating and improving this type of tourism. The information is provided after analyzing how the different factors influence the management and promotion of cultural tourism. There are previous studies on Islamic tourists [44, 45], but few have investigated this topic in Spain [8] and none of them use the American Customer Satisfaction Index (ACSI). There are studies that link heritage and tourism with authenticity and loyalty at a destination [46], motivation and satisfaction [47], perceived value and loyalty [48], experience, satisfaction and tourist behavior [49] and satisfaction and loyalty [50]. However, there are no studies measuring satisfaction, perceived value and real value with loyalty to a heritage site and a mosque for the type of cultural tourists investigated in this document. Different approaches and views [51] have been presented in the literature for the management and administration of cultural assets from a cultural perspective. In order to properly manage and administer this type of tourism, fundamental factors such as the historical Islamic traditions and heritage of the monuments must be taken into account, and in this way tourist loyalty may be increased. This project contributes to the literature on the management and administration of heritage sites by using the opinions and criteria of the community that built and managed the site in the past. Taking these points unto consideration can help to establish and improve management models that achieve the highest levels of loyalty for a historical Islamic heritage site. The following research question is the result of these point—Do the tourist activities available at the Mosque-Cathedral make tourists of Islamic origin feel loyal to the site due to the perceived quality, satisfaction and loyalty as defined in the ACSI model?

## Literature review

### Islamic tourism at mosques

Islamic tourism is generally perceived as a type of tourism encompassing Islam and/or Muslim travelers. Islamic tourism describes Muslim tourists traveling to religious destinations (Organization of the Islamic Conference, 2008). Islam is often perceived as a strict religion which imposes many rules, but it is important to realize that Islam and tourism are compatible. In fact, Islam promotes tourism by mentioning it in several chapters of the Qur’aan (Surah) as a tool for acquiring knowledge. This is why acts of Hajj (pilgrimage to Mecca), Umrah (pilgrimage to Mecca and Medina) and Ziyarat (other religiously motivated trips to mosques, tombs, caves or battlefields) are very important in Islam [52]. The creation of products which respond to the needs of Muslim communities is a key task which involves tangible and intangible elements [53].

There are authors who identify tourism at mosques as an important part of Islamic tourism [54] and as an important type of emerging tourism [55]. In the Muslim religion, mosques are the house of God or places of worship [56]. In fact, the term mosque means prostrate and a mosque is a place where Muslims kneel [57]. and are mainly used for congregational prayers. These buildings are a direct manifestation of Islam and portray Muslim identity [58], representing Islam and its dissemination [59]. In addition, in the Muslim way of life, mosques play important roles in society [58] beyond purely religious ones, such as holding educational, political and community events Aziz, Rahman [59].

Mosque tourism is part of religious tourism and managers and policy makers are showing interest in the promotion of mosques as tourist attractions [60]. In fact, the Mosque-Cathedral of Cordoba was among the top ten destinations for tourists visiting this country in 2017 [61]. Mosques are heritage sites and, as such, are a touristic resource [62]. Overall, academic literature on mosque tourism is very scarce. Studies about this type of tourism are varied, and investigate different areas such as, employment [63], documentation [64], or the management of Muslim heritage [65]. Studies about mosques in Spain have investigated the historical and archaeological aspects, as well as their artistic relevance [66]. Fernández & Ortiz-Cordero, (2020) [67] investigated the arrangement of the columns in the Mosque-Cathedral of Cordoba. Other studies indicate that the Mosque-Cathedral has wonderful acoustics [68] and illumination [69], while others present theories on why the mosque was built [70]. According to Benur & Bramwell (2015) [71], tourist products are being offered in Spain that meets the demands of Muslim tourists [29]. It shows that halal tourism is becoming popular and is growing in countries such as the United Kingdom, Canada, Indonesia and Spain [7, 72, 73].

### Theoretical foundation: Expected and perceived quality, satisfaction and loyalty

The literature on the subject contains studies about the relationships which can exist between a customer and a place or business. The studies show that the customer evaluates and values different aspects of the interaction they have with the place or business and there are different factors which are taken into consideration in the resulting relationship [74, 75]. The customer evaluates many elements of the overall management, which is shown to be important as there is no single factor that independently determines the value a customer gives to a place or business [76]. Analyzing the marketing of the products or services offered by a company is also important as relationships with customers are established and maintained and these relationships can subsequently influence positive results for the company, such as word of mouth promotion and increased sales [76]. Using the literature about the theory of commitment or loyalty, the most important factors in the relationship of the Muslim tourist and a place or business are the expected quality [77], perceived quality [78], satisfaction [79] and motivation [80].

Studies have shown that loyalty to a place is directly related to visitor satisfaction and opinion [81]. Studies analyzing loyalty to a heritage have mainly examined the attitude and intention of the visitor [82]. Studies investigating loyalty to cultural heritage are primarily cognitive and use structural equations to predict destination loyalty [83, 84]. This type of academic studies investigating loyalty can be grouped into two categories, firstly, those which investigate repeated purchases, i.e. tourists returning to a destination, and secondly those which consider loyalty to mean recommending a tourist destination to other future tourists [85–87]. Therefore, tourists can feel connected to a destination, and intend to visit it again in the future and recommend it to third parties [88–90]. However, there are other studies that show that a tourist’s desire for new experiences can counteract how loyal a visitor feels to a place [91].

Aspects such as the comfort experienced by the tourist at the destination and, especially, at the monument visited is one of the most important factors to predict whether a visitor will return to a destination and, therefore, for loyalty [84, 87, 90]. Perceived quality is often considered one of the most important factors of loyalty [92]. Perceived quality is defined as the general accumulation of all the tourist’s feelings about their experience at a destination [93, 94]. The importance of perceived quality is related to its relationship with customer satisfaction [95, 96]. Tourists assess their experience as positive or negative based on the attributes of the monument or destination [97]. As both variables above, perceived quality and customer satisfaction, are positively related the probability that a visitor repeats or recommends the destination is high if they give a positive assessment. This relationship is confirmed in numerous academic studies [20, 98–101]. In the proposed model, perceived quality directly influences loyalty and is mediated through satisfaction [84, 102, 103].

H1: The expectations of the Islamic tourist at the Mosque-Cathedral have a significant and positive relationship with the satisfaction of the visit to the Mosque-Cathedral.

H5: The expectations of the Islamic tourist at the Mosque-Cathedral have a significant and positive relationship with expectations and loyalty (mediated).

Following this idea, Escamilla Santamaría [104] y Duque-Oliva and Rodríguez-Romero [105] together with Fernandez Neto [106] state that loyalty to destinations is influenced by the perceived quality i.e. the feelings a tourist has about a place before they go compared to their feelings about it when there. Kim [107] claimed that the preconceived image of a destination is one of the main factors when attracting visitors, so expectations both from the point of view of creating and promoting places by managers and fulfilling expectations is a difficult task that must be well thought out and prepared to achieve the satisfaction of the tourist. Veasna, Wu [108] o Assaker, Vinzi [109] claim that expectations are precisely one of the critical factors to be considered for visitor satisfaction. In the proposed model loyalty was investigated based on expectations using a mediated variable such as satisfaction [80, 110]. This variable is useful for managers when promoting a destination and can be used to manage a site, as it influences whether a visitor will return to a place or recommend it to others.

H2: The quality of the experience of the Islamic tourist at the Mosque-Cathedral has a significant and positive relationship with loyalty to the Mosque-Cathedral.

H3: The quality of the experience of the Islamic tourist at the Mosque-Cathedral has a significant and positive relationship with the satisfaction of the Islamic tourist’s visit to the Mosque-Cathedral.

H6: The quality of the experience of the Islamic tourist at the Mosque-Cathedral has a significant and positive relationship with loyalty. (mediated).

Satisfaction can be defined as the overall assessment of the service received by tourists compared to the ideas they had about what they expected to receive [111]. This definition is based primarily on the cognitive dimension of satisfaction, but it should be noted that there is also an emotional component involved [112]. Satisfaction is a customer’s evaluation made after receiving a product or service, and shows the relationship between expectations and perception.

H4: The satisfaction of the Islamic tourist at the Mosque-Cathedral has a significant and positive relationship with loyalty to visiting the Mosque-Cathedral.

## Materials and methods

The loyalty of customers is an important aspect to consider because it is directly related to the profitability of products and services [113]. It includes variables that have an impact on the intention of Muslim tourists to visit a cultural heritage site and recommend it to others. To this end, expectations (EXP), loyalty (LOY), satisfaction (SAT) and expected quality (QUA) are analyzed individually. The proposed model for this study uses four variables to measure the loyalty of Islamic tourists to mosques, in particular the Mosque-Cathedral of Cordoba, 1) The perceived quality of the tourist experience at the Mosque-Cathedral. Giving a value to this variable implies that the visit was recent, 2) Expected quality measures what the customer expected to obtain from the visit. The value given to this variable represents any past experience at a mosque [30], 3) Customer satisfaction is a measure that takes into account the number of people who say they will repeat their visit or recommend the destination to others.

The hypotheses used in the empirical investigation are given below. The main objective of this study was to identify the factors that significantly influence the loyalty of Muslim tourists who visit cities with mosques by analyzing six hypotheses formulated from the existing literature. The hypotheses used in this study are listed below:

H1. The expectations of the Islamic tourist at the Mosque-Cathedral have a significant and positive relationship with the satisfaction of the visit to the Mosque-Cathedral.
H2. The quality of the experience of the Islamic tourist at the Mosque-Cathedral has a significant and positive relationship with loyalty to the Mosque-Cathedral.
H3. The quality of the experience of the Islamic tourist at the Mosque-Cathedral has a significant and positive relationship with the satisfaction of the Islamic tourist’s visit to the Mosque-Cathedral.
H4. The satisfaction of the Islamic tourist at the Mosque-Cathedral has a significant and positive relationship with loyalty to visiting the Mosque-Cathedral.
H5. The expectations of the Islamic tourist at the Mosque-Cathedral have a significant and positive relationship with expectations and loyalty (mediated).
H6. The quality of the experience of the Islamic tourist at the Mosque-Cathedral has a significant and positive relationship with loyalty. (mediated).

## Methodology

### Description of the Mosque-Cathedral in Córdoba

The investigation was carried out in Córdoba, which is part of the autonomous community of Andalusia in southern Spain. It is an inland city which is well connected to the rest of the country by means of roads and high-speed trains. It is attractive to tourists due to its wealth of cultural heritage (World Heritage Cities of Spain, 2017). The Mosque-Cathedral, where the field work was carried out, has two distinct areas. The first is the courtyard, where the current bell tower and the prayer room are located. The latter stands out because of the myriad of columns and bi-colored arcades. The second area is the interior of the mosque-cathedral, which is divided into five zones that show the five enlargements carried out over the centuries.

### Questionnaire and scales

The information was collected using a questionnaire together with a personal interview with each tourist of Islamic origin after their visit to the Mosque-Cathedral. The questionnaire was prepared in December 2019. The validation of the survey and the construction of the questions is based on consolidated indicators from previous research [114, 115]. Once the indicators had been obtained, a two-stage refining process was used. First, the indicators proposed by an investigator were analyzed, then the final survey was tested and verified by a manager at the Mosque-Cathedral. This meant that the validity of the indicators in the constructs of the proposed research model were checked twice.

The access by the surveyors to the Mosque-Cathedral heritage and the conduct of interviews with tourists was authorized by the managing body and owner of the Mosque-Cathedral (Cabildo Mosque-Cathedral of Córdoba). Prior to the completion of the questionnaire, tourists were informed of academic purposes and anonymity in answering. Consent to take the questionnaire was verbal. At all times, the visitor’s anonymity to the Mosque-Cathedral was guaranteed.

The questionnaire was organized into six sections. The first section included questions about the tourist’s demographic profile. The following sections asked about the expectations the tourists had for the destination, the quality of the destination, their general experience and their loyalty, which means their intention to return or recommend a visit to the city and/or the Mosque-Cathedral. The different variables were measured on a 5-point Likert scale, where 1 means totally disagree and 5 means totally agree. The previously tested questions from other studies, as shown in Table 1, were adapted and used in this investigation.

**Table 1:** Scales used.

Authors	Variable	Indicators
[60, 116, 117]	Expectations (EXP)	(EXP1) Conservation of the Mosque-Cathedral. (EXP2) Conservation and cleanliness of the city. (EXP3) Conservation and cleanliness of the Mosque-Cathedral.(EXP4) Transport to the city.
[98–120]	Satisfaction (SAT)	(SAT1) The visit to the Mosque-Cathedral was satisfactory. (SAT2) The visit to the Mosque-Cathedral met my expectations. (SAT3) The conduct of the staff at the Mosque-Cathedral was satisfactory. (SAT4) The cultural heritage of the monument was better than expected. (SAT5) The visit to the Mosque-Cathedral is worth the entrance fee. (SAT6) My experience at the Mosque-Cathedral was positive.
[80, 108–110]	Loyalty (LOY)	(LOY1) I would recommend visiting the city of Córdoba to my family and friends. (LOY2) I would recommend visiting the Mosque-Cathedral to family and friends.
[102, 103, 121]	Destination Quality (QUA)	(QU1) Prestige of the city. (QU2) Prestige of the Mosque-Cathedral. (QU3) Conservation of the Mosque-Cathedral. (QU4) Conservation and cleanliness of the city.

Source: Author.

In a second phase the survey was made in English and Spanish. Before starting the questionnaire, the pollster asked the tourist for their cooperation and provided information about the objectives of the investigation. The tourist of Islamic origin completed the survey anonymously with complete autonomy. Indicators about the sociodemographic profile were included at the end of the survey. The questionnaire contained 32 indicators, which were the result of debugging indicators after calculating the value of the Cronbach’s Alpha coefficient for each construct. The fieldwork was carried out during the month of December 2019, with a simple random sampling of visitors to the Mosque-Cathedral. A pretest of 20 surveys was conducted. In total, the number of valid questionnaires was 262, obtaining a 95% confidence level. The target population of the study is 2,079,160 tourists who visited the Cathedral Mosque in 2019. With a sampling error of 6% and a confidence interval of 95%, we obtain a finite representative sample for the population of the entire year 2019 of 262 questionnaires. 270 questionnaires were carried out, 8 of which were discarded due to errors in completion (Table 2).

**Table 2:** Calculation of sample size.

**Population**	2.079.160,00	
**Error:**	6%	
**Confidence:**	95%	
**Sample size:**	262,76	Infinite
**Sample size:**	262	Finite

Source: Author.

From the indications in the work of Nunnally and Bernstein (1994), [122] the value of Cronbach’s alpha for all the indicators was calculated. The value obtained, 0.913, was acceptable, because a value of 0.7 or above is considered acceptable. The data obtained was tabulated and analyzed using the IBM SPSS 23 statistical system (IBM Corporation, Armonk, NY, USA) and the Smart-PLS (Partial Least Squares) structural equation computer package. The literature review showed that the SEM method is considered the most appropriate way to validate the hypotheses proposed in the structural equations and confirm the model of complex relationships. The Smart PLS 3.2.9 program [123], for modeling structural equations with minimum partial squares (PLS-SEM) was used as a tool for the analysis of complex interrelationships between observed and latent variables. This method has been widely used and validated for scientific research in the tourism sector [124].

## Results and discussion

The main findings for each of the variables in the questionnaire are described below. The sociodemographic results were used to define the profile of the tourists surveyed during their visit to Cordoba (see Table 3). Secondly, the reliability and validity of the proposed model are studied, and finally, the hypotheses are analyzed.

**Table 3:** Sociodemographic profile of the respondents.

Variable	Categories	Absolute Frequency	Percentage
**Sex *(n = 262)***			
Men		121	46
Women		141	54
**Age *(n = 262)***			
[Under 25]		79	30
[26–39]		69	26,2
[40–59]		32	12,2
60 or more		82	31,6
**Level of Studies (n = 262)**			
Basic		20	7,6
Secondary		40	15.2
University		108	41.4
Professional Training		51	19,4
Dk/Da		33	12,5
Others		10	3,8

Source: Author.

The sociodemographic profile of the tourists visiting the Mosque-Cathedral is shown in Table 3 and it can be seen that 54% of the interviewed tourists were women and 46% were men. The surveys were mainly answered by people under 25 years old (30%) with university studies (41.4%).

### Reliability and validity of the model

This model shows the theoretical relationships between the research elements. The model was analyzed to find the validity and reliability of the constructs and which are reflective and which formative. The results obtained show that the observed variables measure the theoretical constructs proposed above [125].

The validity and reliability of the construct measurements were then checked before investigating the relationships between them [126]. The constructs in the model were evaluated following the recommendations in the literature [124]. After the evaluation, loyalty was found to be a formative indicator in the proposed model, using the guidelines of Henseler [127]. According to other authors [128, 129], the multicollinearity between the different indicators that make up formative constructs must be checked. The tests for convergent validity were found to be positive after analyzing the redundancy of these constructs [130]. The collinearity was then assessed by calculating the variance inflation factor (VIF) which was found to be less than 5. A value of 5 or less indicates the non-existence of high multicollinearity [131], even though other authors, such as [132] indicate that a value of VIF greater than 3.3 indicates that there is high multicollinearity. The indicators of the formative constructs were evaluated by weighting, and were found to be significant [124] as shown in Table 4. The resulting values in Table 3 show the absence of collinearity in the variables that make up the loyalty construct.

**Table 4:** Reliability of individual indicators (formative).

Construct	Weighting	Collinearity
Actitud ambiental		
LOY1	0,491	1,601
LOY4	0,621	1,601

Source: Author.

In order to evaluate the reflective constructions or B mode of the model (Satisfaction, Expectations and Quality) the individual reliability of the indicators, the internal consistency of the construct, the convergent validity and the discriminant validity of the reflective constructs were all calculated [125]. The results indicated adequate individual reliability, as all load values were above the required minimum threshold of 0.505 [133] or 0,6 [126]. In fact, the analysis revealed that the loads were statistically significant with a value of 99.99%. The discriminatory validity was also verified, as the correlations between constructions were less than the square root of the average variance extracted [130]. The results of these measurements showed that the measurement model could be considered valid and reliable. The following the structural model analysis could then be carried out.

#### Composite reliability (Pc)

The individual reliability of the constructs allows us to check whether the indicators actually measure the constructs. The results in Table 5 indicate that all the constructs are reliable, as their composite or joint reliability is > 0.7. These values are considered "satisfactory to good" because they are between 0.70 and 0.95 [134]. The composite reliability index is similar to Cronbach’s alpha, with the difference that composite reliability is not influenced by the number of indicators on the scale. Following Hair et al. (2012) [125] the accepted level of composite reliability is 0.7, with 0.8 being a stricter minimal level. In our study, composite reliability can be observed in Table 6. The strictest level of 0.8 is exceeded in three composite reliability indicators (satisfaction, expectations and quality). These values therefore validate the internal consistency of the model. The construct of loyalty as a formative indicator does not have any composite reliability, as formative indicators do not co-vary.

**Table 5:** Individual reliability of the indicators (reflective).

Variable	Load
QU1	0,740
QU2	0,738
QU3	0,700
QU4	0,557
EXP1	0,740
EXP2	0,771
EXP3	0,713
EXP4	0,698
SAT1	0,841
SAT2	0,786
SAT3	0,729
SAT4	0,708
SAT5	0,733
SAT6	0,786

Source: Author.

**Table 6:** Composite reliability.

Construct		Composite Reliability
LOY	Loyalty	n/a
SAT	Satisfaction	0,894
EXP	Expectations	0,821
CAL	Quality	0,802

Source: Author.

#### Convergent validity

The convergent validity is used to find the degree to which the indicators measure the related construct. The most common measurement for evaluating convergent validity in PLS-SEM is the average variance extracted, AVE. Using the same theory as for individual indicators, a value for AVE of 50% or more means that, on average, the construct explains more than half of the variance of its own indicator [123, 124, 133, 135]. Table 7 shows the values of AVE for the constructs, all of which have a value greater than 0.5, and so meet the necessary criteria.

**Table 7:** Average variance extracted.

Construct		Average Variance Extracted (AVE)
LOY	Loyaty	n/a
SAT	Satisfaction	0,586
EXP	Experience	0,535
QUAL	Quality	0,504

Source: Author.

The convergent validity is used to find the degree to which the indicators measure the related construct. The most common measurement for evaluating convergent validity in PLS-SEM is the average variance extracted, AVE. Using the same theory as for individual indicators, a value for AVE of 50% or more means that, on average, the construct explains more than half of the variance of its own indicator [123, 124, 133, 135]. Table 7 shows the values of AVE for the constructs, all of which have a value greater than 0.5, and so meet the necessary criteria [133].

#### Discriminant validity

This value shows the extent to which one construct is different from the others. In order to be in an acceptable range, the values present on the diagonal must be significantly higher than those found in the rows and columns. The values of discriminant validity in this study are shown in Table 8. All the values meet the requirements stated above. It must be noted that since the construct with formative indicators has no AVE, it does not have discriminant validity.

**Table 8:** Discriminant validity.

	**EXP**	**LOY**	**QUA**	**SAT**
**LOY**	n/a			
**EXP**	0,732	n/a		
**QUA**	0,876	0,653	0,710	
**SAT**	0,750	0,776	0,804	0,765

Source: Author.

## Verification of the hypotheses

### Explained variance (R^2^)

Once the sample measurement model had been validated, the internal model was evaluated. This was done to verify the proposed assumptions for the relationships between constructs. To do this, the value of R^2^ was calculated so that the importance of the relationships could be analyzed [125]. The explained variance of endogenous constructions was found by analyzing the calculated values for R^2^ and thus, the predictive power of the model could be found [133].

### Bootstrapping

The significance of path coefficients was found using the t-distribution of Student with 499 degrees of freedom (n-1, where n represents the number of subsamples). The values for the significance were found to be 0.007, 0.000 and 0.010. Table 9 shows the relationship studied, the effect that the relationship has, the Path Coefficient, the t-value and whether or not the relationship is supported. It can therefore be seen that there are three supported hypotheses (H3, H4, H6) and three unsupported ones (H1, H2, H5).

**Table 9:** Hypotheses verification.

Hypothesis	Effect	Path Coefficient	t-Value	*p*-Value	¿Supported?
H1: EXP with SAT	+	0,199	0,817	0,414	NO
H2: QUA with LOY	+	0,083	0,633	0,527	NO
H3: QUA with SAT	+	0,629	2,709	0,007	Sí
H4: SAT with LOY	+	0,710	5,714	0,000	Sí
H5: EXP with LOY		0,141	0,828	0,408	NO
H6: QUA with LOY		0,447	2,581	0,010	SI

a = 0,001 (***); a = 0,01 (**); a = 0,05 (*); n.s. = not supported

Source: Author.

**Fig 1:**
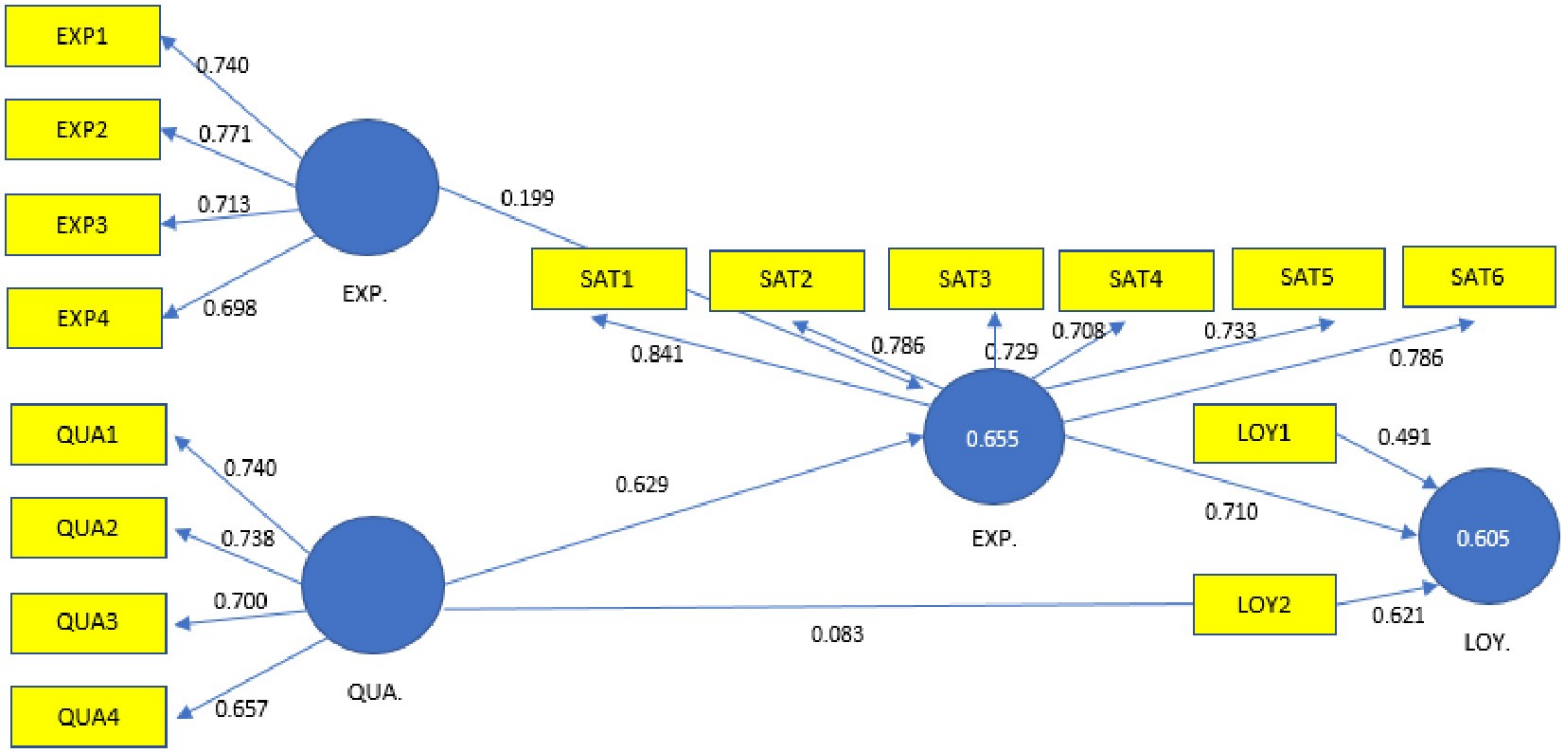
Causal relationships of the proposed model.

The hypothesis H3 (there is a positive relationship between the quality and satisfaction of tourists), hypothesis H4 (there is a positive relationship between satisfaction and loyalty) and hypothesis H6 (there is a positive relationship between quality and loyalty) were all supported. However, hypothesis H1 (customer expectations influence visitor satisfaction), hypothesis H2 (quality influences the loyalty of tourists) and hypothesis H5 (expectations influence loyalty) were not supported in this study. Fig 1 shows the causal relationships of the proposed model.

## Discussion

This study was motivated by the growing importance of tourist loyalty to places visited. In this study the loyalty of Islamic tourists at the Mosque-Cathedral of Cordoba was investigated. The most important aspects for increasing the loyalty of the Islamic tourist at the Mosque-Cathedral were investigated. Córdoba is a city where different cultures have passed and coexisted and the Mosque-Cathedral can be considered as one of the most unique and exceptional monuments in the world. The Mosque-Cathedral is currently one of the heritage sites with the highest number of visits in Spain, surpassing 2 million visits in 2019, with a continuous, average growth of 6.5%. The improvement and increase in the offers of the Mosque-Cathedral, such as the night-time visit ‘Alma de Córdoba’ and the recognition of the value of the Torre Campanario in 2017, have improved the tourist experience and resulted in an increase in overnight stays in the city. It is an example of responsible and sustainable management with the clear aim of growth. These factors, together with the high levels of quality and satisfaction found in this study make it the place with the highest tourist loyalty in the city. All these aspects can be used by managers of historical heritage to make sustainable tourist offers which allow for growth. The opinions of Islamic tourists about their visit helped to identify the factors that influence loyalty, such as satisfaction, quality and expectations in order to identify which were most decisive for the study. Managers responsible for planning and managing important tourist destinations for Islamic tourists can use these factors to increase tourist loyalty and can take appropriate measures implement them. Tourists who are loyal also have high expectations for the quality of the service provided, are satisfied with high quality service and the tourist experience. These tourists will recommend the destination to third parties and intend to repeat their visit. It is advisable to create personalized marketing strategies to ensure the loyalty of this type of tourist [136].

The ACSI model was used in this study to investigate the loyalty of tourists visiting mosques, which could play an important role in the future of the cultural tourism experience in Spain. A structural equation was proposed, that was used to study the loyalty of this type of tourists due to the quality and satisfaction the tourists felt with their visit to the Senarath site [137] Tourists visiting mosques have special characteristics which must be taken into account when managing heritage sites [138]. This means that the heritage sites that this type of tourists visit should read and use academic studies for the organization and management of the site, as well as for planning tourism at the site. As a result of the problems arising from the mismanagement of heritage sites, group identity can be lost and stereotypes can be created [33, 139] which can have negative effects on tourism at the site.

According hypothesis 3, there is a significant and positive relationship between quality and satisfaction. A high level of expected quality is expected to have a positive effect on the value given by tourists to perceived quality [108, 109]. Expected and perceived quality are the basic requirements for any successful tourist destination. The local community plays an important role in the feelings of tourists at a destination. According hypothesis 2, the quality of service gives a value to the experience and feelings of the tourist. This aspect may influence the tourist’s intention to revisit or recommend a destination or heritage site. The perceived quality index value (87.89%) exceeded the expectations index value (86.19%) of the tourists in this study. This is consistent with results from other studies in the field of tourism [119, 140]. According hypothesis 3, it has been shown that trust and quality of service have a positive and significant effect on customer satisfaction and that, in turn, satisfaction has a positive influence on customer loyalty (hytpothesis 4) [115, 116]. There are studies which confirm this relationship for the Islamic collective in Malaysia [141] for areas of tourism such as heritage, halal, alcohol, free gambling and Islamic morality [142]. These outcomes are in line with other studies that analyze consumer behavior and culture [116]. In recent years, managers at the Mosque-Cathedral have been concerned about improving the quality of the tourist experience at this historical heritage site. This can be seen by the reforms made and the new tourism products available at the Mosque-Cathedral. There is also a significant and positive relationship between satisfaction and loyalty (hypothesis 4). According to the results achieved in other studies [20, 97, 101] found that positive feelings about satisfaction, quality and expectations positively influenced intentions to return to or recommend a destination (Hypothesis 5,6). These results are a practical goal for managers to improve the experience of the Islamic tourists visiting the Mosque-Cathedral. This means that satisfaction with the tourist visit becomes one of the reasons for visiting the heritage site. Similarly, and in line with previous work [98, 143] results indicate that satisfaction has a positive influence on loyalty to the destination, which encourages tourists to return to the destination in the future, and to recommend it once they return home. In addition, there is a significant and positive mediated relationship between quality and loyalty. This confirms other studies [144, 145]. In the case of the Mosque-Cathedral improving the quality felt by tourists at the site can also improve the quality and satisfaction felt with the tourist destination.

The main reason why hypotheses 2, 3 and 5 have not been fully fulfilled is based on the idiosyncrasies of this type of tourism itself. The results obtained coincide with studies that analyze this type of tourism and its relationship with the factors analyzed. Following this idea, the motivation of the tourist can influence the results related to satisfaction and loyalty. When the motivation of the visit is based on religious factors both the expectations and the quality expected of the visit go to the background (Idris, 2019; Piva et al, 2019) since the essential thing for the tourist with religious motivations is the visit itself to the place of worship altering the results of our hypotheses.

## Conclusion and limitations

The results show that tourists appreciate different parts of their experience, some of which are, the explanations given by the guides, the conservation of the heritage site, and the care and maintenance of the surrounding areas. The Mosque-Cathedral in Cordoba therefore unites and links the tourist with the destination, positively influencing loyalty to the city. The quality attributed to this heritage site, linked to the perceived quality of the visit is a positive attribute for those travelers who want to know the local culture [110, 146].

Studies which investigate the loyalty of Islamic tourists at heritage sites is scarce [147, 148]. However, the analysis of mosque tourism is becoming important [6, 149]. This study has important theoretical and practical implications for the management of Islamic tourism at heritage sites. First, measuring the loyalty of Islamic tourists is a critical step in designing and implementing practices that improve loyalty. In order to promote the tourist loyalty of this type of tourist, the agents involved must take into account the expectations of the tourists who visit the site, the perceived level of quality of the service, the satisfaction of the visitors and their intention to recommend the destination and repeat the visit. Studying this type of tourist means that the results can be used to prepare specialist marketing strategies for them [150]. Personalized programs for Islamic tourists can be organized, so that stable long-term relationships are established which are interesting for this type of tourist so that their satisfaction with the visit is increased. These are special customers for which personal relationships must be developed. Understanding how the ACSI model works with cultural tourism can contribute to improving the loyalty of Islamic tourism. From a practical point of view, the results corroborate the idea that the correct management of the heritage site can influence the feelings of the Islamic tourist, which increases their loyalty to the site and affects the recommendations they make. This means that the tourism sector should take these factors into account to improve tourism loyalty. Policymakers should explore new management methods to give this type of tourist a memorable experience when they visit heritage sites. The management of information, the preservation of the site, its prestige and the safety felt when visiting are all important points to consider. To avoid possible disagreements, the management of these heritage sites should pay particular attention to Islamic identity and its relationship to the site, including the religious and institutional orientation. Another possible area of conflict could be non-Islamic interest in mosques, because public authorities, operators and tourists themselves may have no special interest in Islamic heritage. The local tourist board has a new system which promotes this type of tourism, which can be considered a tourist niche [129, 130] in order to support a multicultural and cosmopolitan city. Local government and private companies which are responsible for the management of the Mosque-Cathedral should work together to improve the tourism experience [129–131]. Specialist communication tools can play a key role as marketing strategies to attract this type of tourism.

This research has potential limitations, the first of which is the sample used. The data was only obtained from tourists in the Mosque-Cathedral in Cordoba, which could indicate that the collected data is only applicable to one unique tourist site. Another improvement could be a longitudinal study that records the feelings of tourists over time, allowing measurement of variables in a more precise way. Also, although measuring behavioral loyalty has been used in several studies, it is a subjective measure of tourist behavior and does not always correspond to tourists’ real behavior [132]. Future lines of research could include other internal and external variables, as well as studying of the relationships between destiny, heritage and loyalty.

## Supporting information

S1 Data (XLSX)
